# Olive Oil Extracts and Oleic Acid Attenuate the LPS-Induced Inflammatory Response in Murine RAW264.7 Macrophages but Induce the Release of Prostaglandin E2

**DOI:** 10.3390/nu13124437

**Published:** 2021-12-11

**Authors:** Anke Katharina Müller, Franziska Albrecht, Carsten Rohrer, Andreas Koeberle, Oliver Werz, Wiebke Schlörmann, Michael Glei, Stefan Lorkowski, Maria Wallert

**Affiliations:** 1 Department of Nutritional Biochemistry and Physiology, Institute of Nutritional Science, Friedrich Schiller University Jena, 07743 Jena, Germany; anke.katharina.mueller@uni-jena.de (A.K.M.); franziska.albrecht91@outlook.de (F.A.); carsten.rohrer@uni-jena.de (C.R.); stefan.lorkowski@uni-jena.de (S.L.); 2 Competence Cluster for Nutrition and Cardiovascular Health (nutriCARD), Halle-Jena-Leipzig, 07743 Jena, Germany; wiebke.schloermann@uni-jena.de (W.S.); michael.glei@uni-jena.de (M.G.); 3 Center for Molecular Biosciences Innsbruck (CMBI), Michael Popp Institute, University of Innsbruck, 6020 Innsbruck, Austria; andreas.koeberle@uibk.ac.at; 4 Department of Pharmaceutical/Medicinal Chemistry, Institute of Pharmacy, Friedrich Schiller University Jena, 07743 Jena, Germany; oliver.werz@uni-jena.de; 5 Department of Applied Nutritional Toxicology, Institute of Nutritional Science, Friedrich Schiller University Jena, 07743 Jena, Germany

**Keywords:** olive oil, oleic acid, inflammation, macrophages, phospholipid incorporation

## Abstract

Olive oil contains high amounts of oleic acid (OA). Although OA has been described to inhibit inflammatory processes, the effects of olive oil on cellular mechanisms remain poorly understood. Therefore, we compared the effects of major fatty acids (FA) from olive oil with those of olive oil extracts (OOE) on inflammatory mediators and alterations in the cellular phospholipid composition in murine macrophages. Upon treatment with different OOE, FA compositions of lipopolysaccharide (LPS)-stimulated murine RAW264.7 macrophages were analyzed using gas chromatography. Olive oil extracts and OA significantly reduced the LPS-induced expression of inducible nitric oxide synthase (iNos), cyclooxygenase (Cox2), and interleukin-6 mRNA. In addition, a significant decrease in Cox2 and iNos protein expression was observed. The formation of nitric oxide was significantly reduced, while the formation of prostaglandin (PG) E_2_ from arachidonic acid significantly increased after treatment with OOE or OA. The latter was associated with a shift in the phospholipid FA composition from arachidonic acid to OA, resulting in an elevated availability of arachidonic acid. Together, OOE and OA mediate anti-inflammatory effects in vitro but increase the release of arachidonic acid and hereinafter PGE_2_, likely due to elongation of OA and competitive incorporation of fatty acids into membrane phospholipids.

## 1. Introduction

The Mediterranean diet is characterized by high consumption of vegetables, fruits, nuts, and particularly extra virgin olive oil (EVOO) and has been proposed to be protective against a broad range of diseases [1,2,3]. Especially the association between cardiovascular diseases and atherosclerosis, a chronic inflammatory disease, has been widely studied [4,5].

During inflammation, macrophages play a pivotal role by secreting both pro- and anti-inflammatory mediators [6]. The aim of the inflammatory reaction is the elimination of the initial cause itself and the restoration of physiological homeostasis. Within the inflammatory response, numerous regulatory factors participate that promote or resolve inflammation [7]. Typical immunomodulatory signaling molecules released by activated macrophages are cytokines, such as proinflammatory tumor necrosis factor (Tnf) α, interleukin (Il) 1β and 6, and anti-inflammatory Il10 [8,9]. Inflammatory stimuli lead to an activation of cyclooxygenase (Cox) 2 and inducible nitric oxide synthase (iNos) [10,11]. While the inducible isoform Cox2 generates proinflammatory eicosanoids, such as prostaglandin (PG) E_2_, iNos catalyzes the formation of the second messenger nitric oxide.

Beneficial effects of the Mediterranean diet may be partially mediated by the high intake of EVOO, with its high amounts of antioxidants and the monounsaturated fatty acid (MUFA), oleic acid (OA) [12]. OA and antioxidants inhibit endothelial activation [13] and reduce the proinflammatory response induced by lipopolysaccharide (LPS) in murine microglial cells, murine macrophages, and murine bone marrow-derived dendritic cells [14,15,16]. In rats, OA is the responsible factor for the hypotensive effects of olive oil consumption [17]. Since anti-inflammatory effects of OA on cellular processes are well described, and OA is a major component of olive oil, we hypothesize that olive oil itself and especially its lipophilic fraction have similar effects on inflammatory processes.

Therefore, we here investigated the effects of an olive oil extract (OOE), prepared from a commercial EVOO, on the inflammatory response in LPS-induced murine RAW264.7 macrophages and compared its activity to OA. Furthermore, we analyzed the effects of EVOO and OA on the FA composition of membrane phospholipids (PL) to draw conclusions on cellular uptake and incorporation.

## 2. Materials and Methods

### 2.1. Chemicals and Samples

Unless indicated otherwise, chemicals were purchased from Carl Roth (Karlsruhe, Germany), Sigma-Aldrich (Seelze, Germany), Thermo Fisher Scientific (Schwerte, Germany), or Merck Millipore (Darmstadt, Germany). Five commercially EVOOs available in German supermarkets were used as a representative of typical dietary olive oils in Germany.

### 2.2. Saponification of Olive Oils

As previously described [18], fatty acids were hydrolyzed from triglycerides using chemical saponification. The quality of dissociation was verified by thin-layer chromatography [17].

### 2.3. Analysis of Fatty Acids by Gas Chromatography (GC)

Fatty acid methyl esters (FAME) were obtained from the triglycerides of EVOOs using *n*-hexane, and sodium methylate for transesterification. To analyze FA ranging from 4 to 26 carbon atoms, a GC-FID (GC-17 V3; Shimadzu, Kyoto, Japan) was used. The FAMEs were separated on a fused-silica capillary column DB-225ms (60 m × 0.25 mm, i.d. with 0.25 µm film thickness; J&W Scientific, Agilent, Santa Clara, CA, USA) using H_2_ as carrier gas (total flow rate 45 mL/min). A GC oven temperature program was employed as follows: start at 70 °C, held 2 min, 70–180 °C, ramp 10 °C/min (11 min), 180–220 °C, ramp 2 °C/min (20 min), held 5 min, 220–230 °C, ramp 2 °C/min (5 min), held 27 min, total run time 70 min. Fatty acid concentrations are expressed as a percentage of the total area of all FAME (% of total FAME). For quantification, GC Lab Solution software version 2.3 (Shimadzu, Jena, Germany) was used. The total concentration of OOE was calculated with respect to the molecular masses of the individual FAs and the mean density of olive oil [19] (Supplementary Materials Table S1).

For analyzing FA distribution in vitro, cells were harvested in phosphate-buffered saline (PBS); total cellular fat was extracted with a methanol/chloroform mixture based on the protocol by Bligh and Dyer [20] and separated using thin-layer chromatography. Fractions of interest were scraped off from thin-layer chromatography plates. In the following, sodium methylate was used for transesterification of PLs to FAME.

### 2.4. Coupling of Fatty Acids

Bovine serum albumin (BSA) served as a vehicle for free FAs [21]. Fatty acids and saponified OOEs were diluted in a mixture of BSA and Krebs Ringer bicarbonate buffer to a ratio of 4:1 (FA/BSA) [22] as previously described [18].

### 2.5. RAW264.7 Macrophage Culture

Murine RAW264.7 macrophages (ATCC, Manassas, VA, USA) were cultivated as described [23]. Cells were seeded to a confluent cell layer in 24-well plates, cultured for 24 h, and further treated as indicated in the figures. Cells were harvested for further processing as described below.

### 2.6. Cell Viability

Cell viability of RAW264.7 cells was determined using 3-(4,5-dimethylthiazol-2-yl)-2,5-diphenyltetrazolium bromide (MTT; Amresco, Solon, OH, USA) as described earlier [18]. After 24 h of incubation with BSA-coupled OOE or OA (0–200 µM) and washing, MTT solution (0.2 mg/mL) was added. Cells were incubated for 4 h at 37 °C. Next, the cell culture medium was discarded before the intracellularly formed formazan was dissolved in isopropanol. A FLUOstar Omega microplate reader (BMG Labtech, Ortenberg, Germany) was used to measure optical density at 570 nm. Viability units were normalized to the untreated control.

### 2.7. RNA Isolation and cDNA Synthesis

RNA isolation and cDNA synthesis were performed as described [18,24].

### 2.8. Quantitative Real-Time RT-PCR

RT-qPCR analyses were performed as described elsewhere [18,25]. Primers (Il6, Il10, Il1β, Tnfα, iNos, Cox2, and peptidylprolyl isomerase B (Ppib)) were purchased from Invitrogen (Karlsruhe, Germany; Supplementary Materials Table S2). Results were analyzed using LightCycler software version 1.5.0.39 (Roche Applied Science, Mannheim, Germany). Ppib was used as a reference gene. The fold change of mRNA expression was normalized to the expression of Ppib. Quantitative analysis was performed using the 2-ΔΔCT method.

### 2.9. Immunoblotting

A non-denaturing buffer was used for harvesting cells as previously described [23]. Proteins were separated by SDS-PAGE and transferred to a PVDF membrane (Carl Roth, Karlsruhe, Deutschland). Anti-iNos and anti-Cox2 antibodies were diluted at 1:2000 and 1:10,000, respectively, with signal enhancer solution (SignalBoost^TM^ Immunreaction Enhancer kit, Merck KGaA, Darmstadt, Germany). Antibodies were used as described elsewhere [18]. For detection, Pierce ECL Western Blotting Substrate and CL-XPosureTM Films (Thermo Fisher Scientific, Waltham, MA, USA) were applied. Blots were analyzed densitometrically using ImageJ software version 1.43u (U.S. National Institutes of Health, Bethesda, MD, USA). Relative expression was normalized to α-tubulin.

### 2.10. Quantification of Nitric Oxide Formation Using Griess Assay

Supernatants of RAW264.7 cells were treated as described previously [26]. Nitrite concentration was measured at λ = 544 nm with a FLUOstar Omega microplate reader and analyzed as described [18].

### 2.11. Analysis of PGE_2_ and Arachidonic Acid (AA) by UPLC-MS/MS

Prostanoids and polyunsaturated fatty acids were extracted from cell supernatants using Sep-Pak C18 35 cc Vac Cartridges (Waters) and separated on an Acquity UPLC BEH C18 column (1.7 µm, 2.1 × 50 mm; Waters, Milford, MA, USA) using an AcquityTM UPLC system (Waters, Frankfurt, Germany) coupled to a QTRAP 5500 Mass Spectrometer (Sciex, Darmstadt, Germany) [23,27]. Analytes were ionized in negative ESI mode and quantified by multiple reaction monitoring (AA: *m*/*z* 303 → 259; PGE_2_:351 → 271). Mass spectrometric parameters were set as follows: ion spray voltage, −4000 V; heated capillary temperature, 500 °C; sheath gas pressure, 40 psi; auxiliary gas pressure, 40 psi; declustering potential, −120 eV (PGE_2_) or −100 eV (AA); collision energy (−20 eV for PGE_2_,−16 eV for AA). Signal intensities from automatic peak integration (Analyst 1.6, Sciex, Framingham, MA, USA) were normalized to the internal standard PGB_1_ (335 → 113) using IntelliQuan software (version, Sciex, Framingham, MA, USA) default settings. In variation to the method described above, the cell culture medium was cleaned by precipitating proteins in methanol for the analysis of polyunsaturated fatty acids [28,29]. The supernatants were acidified and extracted on Sep-Pak C18 6cc Vac Cartridges (Waters, Milford, MA, USA) and subjected to UPLC-MS/MS analysis. Chromatographic conditions and mass spectrometric parameters were adjusted as described previously [28,29]. AA concentrations were calculated based on the internal standard d8-AA (Cayman Chemicals, Ann Arbor, MI, USA) in combination with external calibration (R^2^ = 0.999; LOQ = 25 ng/mL, defined as signal/noise ratio >10).

### 2.12. Statistics

Data are presented as means ± standard deviations of at least three independent experiments. Statistical differences were analyzed by one-way ANOVA, including Tukey post-hoc test or ordinary one-way ANOVA, including Dunnett post-hoc test using GraphPad Prism for Windows version 7 (GraphPad Software, San Diego, CA, USA). For all statistical analyses, *p* < 0.05 was considered statistically significant.

## 3. Results

We comparatively studied the effects of an OOE, consisting of the lipophilic fraction of EVOO and FAs, on the inflammatory response in LPS-induced RAW264.7 macrophages. To exclude cytotoxic effects in the following experiments, we treated cells with OOE and OA and determined cell viability. OOE and OA did not significantly affect cell viability up to concentrations of 200 µM (Supplementary Materials Table S3).

### 3.1. OOE and OA Inhibit the LPS-Induced Expression of Target Genes in RAW264.7 Macrophages

**Figure 1:**
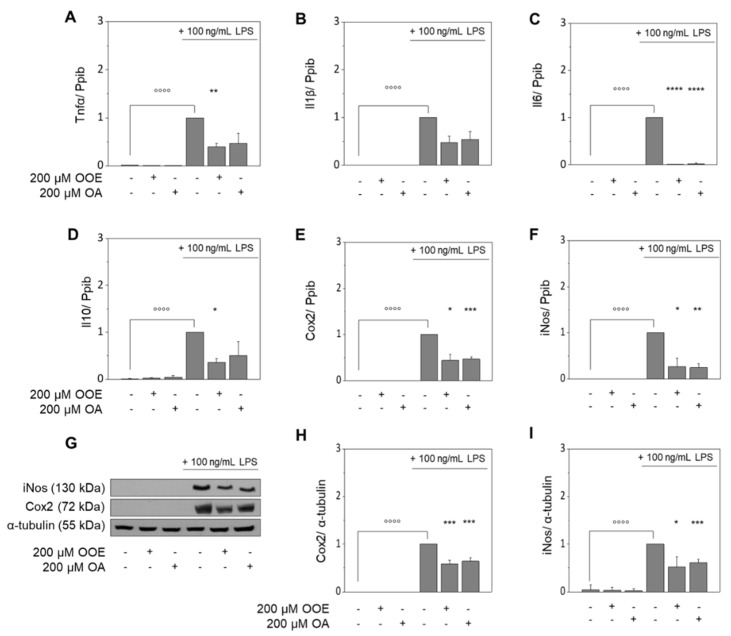
Effects of olive oil extract (OOE) and oleic acid (OA) on the expression of inflammatory markers. Extracts of olive oil and OA diminish LPS-induced expression of Tnfα, Il1β, Il6, and Il10 mRNA (**A**–**D**), as well as iNos and Cox2 mRNA and protein expression (**E**–**I**) in murine RAW264.7 macrophages. Cells were pre-incubated with medium, 200 µM OOE or 200 µM OA for 4 h followed by co-incubation with 100 ng/mL LPS for an additional 20 h. Untreated control samples were cultured with serum-free medium for 4 h plus 20 h of serum-free medium; LPS-treated samples were defined as 1. Expression of mRNA and protein was normalized to Ppib or α-tubulin, respectively. Western blots shown here are representative examples of the blots used for densitometry. Data are shown as mean expression levels ± standard deviation of four (**A**–**F**) or six (**H**,**I**) independent biological replicates. Significant differences compared to the untreated control (°°°° *p* < 0.0001) and to the LPS control (* *p* < 0.05; ** *p* < 0.01; *** *p* < 0.001; **** *p* < 0.0001) were obtained by repeated-measures (RM) one-way ANOVA tests.

LPS significantly induced mRNA expression of proinflammatory cytokines (Tnfα, Il1β, Il6) and elevated Cox2 and iNos mRNA and protein levels in RAW264.7 cells as compared to non-stimulated cells (Figure 1A–I). LPS-treated samples were set to 1, which defines 100%. While the LPS-induced expression of Tnfα was significantly reduced to 39% ± 7% (*p* < 0.01) by OOE and 47% ± 21%, even though just tendentially (*p* = 0.07), by OA (Figure 1A), the effects for Il1β tended to be blocked to 47% ± 14% (*p* = 0.08) by OOE and to 54% ± 16% (*p* = 0.14) by OA (Figure 1B). For both conditions, LPS-induced Il6 expression was significantly blocked to basal levels (*p* < 0.0001) (Figure 1C); just the treatment with OOE significantly decreased LPS-induced expression of Il10 to 36% ± 8% (*p* < 0.05; Figure 1D). Due to an interference with mRNA expression (Cox2: OOE: 44% ± 14%, *p* < 0.05; OA: 47% ± 4%, *p* < 0.001; Figure 1E; iNos: OOE: 27% ± 18%, *p* < 0.05; OA: 25% ± 9%, *p* < 0.01; Figure 1F), both, OOE and OA, significantly lowered the LPS-induced protein expression of Cox2 to 58% ± 8% and 64% ± 7%, respectively (*p* < 0.001; Figure 1G,H) and of iNos to 52% ± 22% (*p* < 0.05) and 61% ± 7%, respectively (*p* < 0.001; Figure 1G,I).

### 3.2. Fatty Acids of EVOO Inhibit the LPS-Induced Expression of Target Genes in RAW264.7 Macrophages

**Figure 2:**
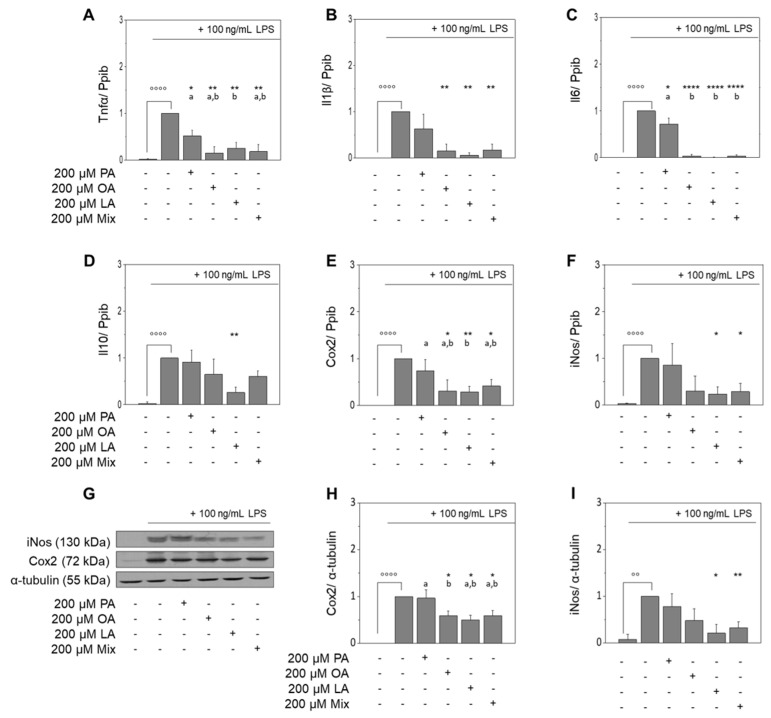
Effects of major fatty acids (FA) of extra virgin olive oil on the expression of inflammatory markers. Selected FAs diminished LPS-induced expression of Tnfα, Il1β, Il6, and Il10 mRNA (**A**–**D**) and of iNos and Cox2 mRNA and protein expression (**E**–**I**) in murine RAW264.7 macrophages. Cells were pre-incubated with serum-free medium, oleic acid (OA), linoleic acid (LA), palmitic acid (PA), or a defined FA mixture (Mix) of the mentioned FAs (80:10:10) for 4 h followed by a co-incubation with 100 ng/mL LPS for additional 20 h. Control samples were cultured with medium only; values of positive controls (LPS) were defined as 1. Expression of mRNA and protein was normalized to Ppib or α-tubulin, respectively. Western blots shown here are representative examples of the blots used for densitometry. Data are shown as mean expression levels ± standard deviation of four independent biological replicates. Significant differences compared to the untreated control (°° *p* < 0.01, °°°° *p* < 0.0001), to the LPS control (* *p* < 0.05; ** *p* < 0.01; *** *p* < 0.001; **** *p* < 0.0001) and between samples (^a,b^ *p* < 0.05; different small letters represent statistically different values) were obtained by RM one-way ANOVA tests.

Fatty acids are known to modulate the LPS-induced activation of macrophages [14,15]. Notably, palmitic acid (PA), OA, and linoleic acid (LA) attenuate the expression of LPS-induced inflammation in our experimental setup (Figure 2). However, we were also interested in the effects mediated by a mixture of OA, LA, and PA according to their common proportion in EVOO, i.e., 80:10:10 (Supplementary Materials Table S4), on the LPS-induced inflammatory response in murine RAW264.7 macrophages compared to the individually added FA. We found inhibitory effects on the LPS-triggered induction of Tnfα mRNA expression for PA (52% ± 13%, *p* < 0.05), OA (15% ± 14%, *p* < 0.01), LA (26% ± 13%, *p* < 0.01), and the mixture of these FAs (18% ± 15%, *p* < 0.01; Figure 2A). PA had no significant effect on the LPS-induced mRNA expression of Il1β (Figure 2B), while it was significantly reduced by OA, LA, and the FA mixture to 15% ± 15%, 6% ± 5%, and 17% ± 13%, respectively (*p* < 0.01 for all changes). While all the tested FAs and their mixture significantly reduced the LPS-upregulated expression of Il6 mRNA (72% ± 13%, *p* < 0.05; 4% ± 3%, *p* < 0.0001; 1% ± 0.4%, *p* < 0.0001; 3% ± 3%, *p* < 0.0001; Figure 2C), only LA significantly inhibited the LPS-induced upregulation of Il10 mRNA to 26% ± 11% (*p* < 0.01; Figure 2D). Next to the cytokines, OA, LA, and the FA mixture significantly reduced LPS-induced expression of Cox2 mRNA to 31% ± 23% (*p* < 0.05), 29% ± 12% (*p* < 0.01), and 41% ± 14% (*p* < 0.05), respectively (Figure 2E). In addition, LA, and the FA mixture reduced LPS-induced expression of iNos mRNA to 24% ± 15% (*p* < 0.05), and 29% ± 18% (*p* < 0.05), respectively (Figure 2F). Expression of Cox2 protein in LPS-stimulated macrophages was attenuated by OA, LA, and the FA mixture to 59% ± 10% (*p* < 0.05), 50% ± 10% (*p* < 0.05), and 59% ± 11% (*p* < 0.05), respectively (Figure 2G,H). In addition, the upregulation of iNos protein by LPS was significantly decreased by LA and the FA mixture to 21% ± 19% (*p* < 0.05) and 32% ± 13% (*p* < 0.01), respectively (Figure 2G,I).

### 3.3. Regulation of Nitric Oxide Formation in Murine RAW264.7 Macrophages Depends on Fatty Acid Saturation and Is Dose-Dependent

**Figure 3:**
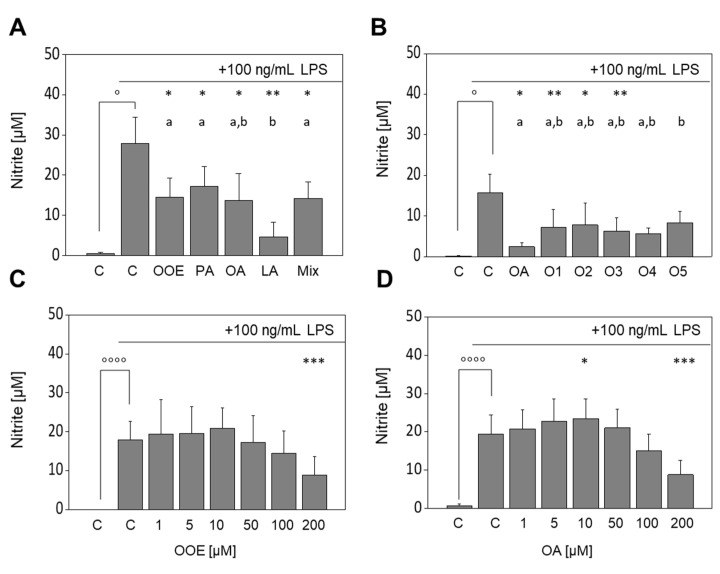
Effects of fatty acids (FA) and olive oil extracts (OOE) on the LPS-induced formation of nitric oxide. Release of nitric oxide measured as nitrite using the Griess assay in LPS-stimulated RAW264.7 macrophages was differentially affected by OOE (O1 to O5) and FA. Cells were pre-incubated with serum-free medium, OOE, oleic acid (OA), linoleic acid (LA), palmitic acid (PA), or of a defined FA mixture (Mix) of the mentioned FAs (80:10:10) for 4 h followed by a co-incubation with 100 ng/mL LPS for additional 20 h (**A**). Cells were pre-incubated with serum-free medium, 200 µM of the five different OOEs (O1 to O5) or 200 µM OA for 4 h followed by a co-incubation with 100 ng/mL LPS with medium, 200 µM OOEs or 200 µM OA for an additional 20 h (**B**). Cells were pre-incubated with medium, 1–200 µM OOE (**C**), or 1–200 µM OA (**D**) for 4 h followed by a co-incubation with 100 ng/mL LPS, 1–200 µM OOE, or 1–200 µM OA for additional 20 h. Untreated control cells were cultured with medium for 4 h plus 20 h. Data are shown as mean formation levels ± standard deviation of 4 (**A**,**B**), 10 (**C**), or 12 (**D**) independent biological replicates. Significant differences compared to the untreated control (° *p* < 0.05, °°°° *p* < 0.0001), to the LPS control (* *p* < 0.05, ** *p* < 0.01, *** *p* < 0.001), and between samples (^a,b^ p < 0.05; different small letters represent statistically different values) were obtained by RM one-way ANOVA tests.

**Figure 4:**
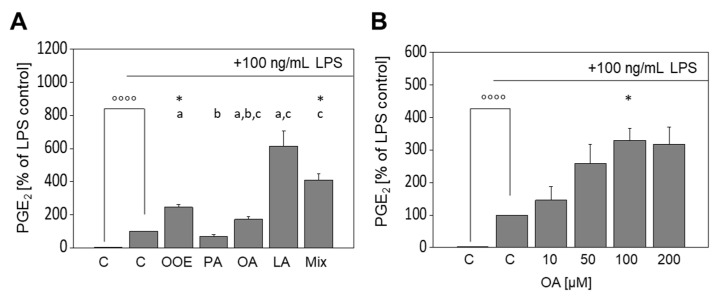
Effects of fatty acids (FA) and olive oil extracts (OOE) on the LPS-induced formation of prostaglandin E_2_ (PGE_2_). The release of PGE_2_ in LPS-stimulated RAW264.7 macrophages was increased by OOE and FAs. Cells were pre-incubated with serum-free medium, OOE, oleic acid (OA), linoleic acid (LA), palmitic acid (PA), or of a defined FA mixture (Mix) of the mentioned FAs (80:10:10) for 4 h followed by a co-incubation with 100 ng/mL LPS for an additional 20 h (**A**). Cells were pre-incubated with serum-free medium or 1–200 µM OA for 4 h followed by a co-incubation with 100 ng/mL LPS for an additional 20 h (**B**). Untreated control samples were cultured with serum-free medium for 4 h plus another 20 h; values of positive controls (LPS) were defined as 100%. Data are shown as mean formation levels ± standard deviation of four (**A**) or three (**B**) independent biological replicates. Significant differences compared to the untreated control °°°° *p* < 0.0001), to the LPS control (* *p* < 0.05) and between samples (^a,b,c,^ *p* < 0.05; different small letters represent statistically different values) were obtained by RM one-way ANOVA tests.

To confirm the anti-inflammatory effects of FAs and the OOE on iNos mRNA and protein expression at the functional level, the production of nitric oxide (NO) by FA- or OOE-treated RAW264.7 macrophages was measured in respective cell culture supernatants. Basal formation of nitric oxide in murine macrophages was negligible (<2 µM; Figure 3), whereas treatment with LPS significantly increased the production of nitric oxide to 27.8 ± 6.6 µM (*p* < 0.05). Co-incubation of LPS with OOE, PA, OA, and the FA mixture significantly reduced the LPS-induced formation of nitric oxide (14.5 ± 4.7 µM, 17.3 ± 4.9 µM, 13.7 ± 6.7 µM, and 14.2 ± 4.1 µM, respectively, *p* < 0.05), and LA showed the strongest effect (4.7 ± 3.6 µM, *p* < 0.01; Figure 3A). To test the consistency of the diminished LPS-induced nitric oxide formation by OOE, five different extracts in Germany’s commercially available EVOO were tested using the Griess assay (Figure 3B). The oils were previously characterized by GC in their fatty acid composition (Supplementary Materials Table S4; O2 represents OOE in Figure 1, Figure 2, Figure 3 and Figure 4). Treatment with LPS significantly increased the formation of nitric oxide (15.7 ± 4.5 μM, *p* < 0.05); the effect of LPS was significantly diminished by OA (2.4 ± 0.9 μM, *p* < 0.05) and OOEs O1 (7.1 ± 4.5 µM, *p* < 0.01), O2 (7.8 ± 5.5 µM, *p* < 0.05), and O3 (6.3 ± 3.3 µM, *p* < 0.01). Olive oils O4 and O5 tended to decrease the LPS-induced production of nitric oxide to a similar extent: 5.6 ± 1.4 μM (*p* = 0.08) and 8.4 ± 2.7 μM (*p* = 0.07), respectively. Significant differences between the different OOEs were not observed. Concentration-response studies revealed a slight upregulation of nitric oxide formation for lower concentrations (10 µM OA: 23.4 ± 5.2 μM nitrite (*p* < 0.05)), whereas higher concentrations reduced the LPS-induced formation of nitric oxide down to 8.8 ± 4.9 µM (OOE) and 8.7 ± 3.8 µM (OA) at 200 µM (*p* < 0.001; Figure 3C,D).

### 3.4. Formation of PGE_2_ in LPS-Stimulated Murine Macrophages Is Augmented by OOE and FA

To explore the effects of FA and the OOE on Cox2 mRNA and protein expression at the functional level, the formation of PGE_2_ by Cox2 in FA- or OOE-treated RAW264.7 macrophages was analyzed in respective cell culture supernatants. The OOE and OA had no effect on PGE_2_ formation in unstimulated cells (data not shown) and LPS significantly induced the formation of PGE_2_ (Figure 4A; *p* < 0.0001). Interestingly, OOE and the FA mixture augmented the LPS-induced formation of PGE_2_ to 244.6% ± 32.5% (*p* < 0.05) and 410.2% ± 71.2% (*p* < 0.05), respectively (Figure 4A), while OA and LA showed non-significant trends to augmented increases of 171.0% ± 31.7% (*p* = 0,11) and 614.2% ± 181.0% (*p* = 0.06). The saturated FA (SFA) PA showed no significant effect. Then, we studied whether OA concentration-dependently augmented the LPS-induced formation of PGE_2_; OA tended to augment the LPS-mediated increase in PGE_2_ to 145.6% ± 42.4% already at 10 µM and significantly enhanced PGE_2_ formation to 329.6% ± 36.2% at 100 µM (*p* < 0.05, Figure 4B), with no further increase at 200 µM.

### 3.5. Oleic Acid Changes the Composition of Cellular Lipids and Phospholipids in RAW264.7 Macrophages

While the inhibition of the LPS-mediated induction of iNos mRNA and protein expression and the decreased LPS-induced formation of nitric oxide were consistent, the augmented release of COX-derived PGE_2_ shown in Figure 4 was unexpected. Arachidonic acid (AA), a common FA in membrane PL, is the precursor of PGE_2_. We, therefore, speculated whether the incubation of RAW264.7 macrophages with OA alters the FA distribution within the cellular lipids. As expected, we observed a concentration-dependent increase in the relative amounts of OA to 40.2% ± 9.8% (100 µM; *p* < 0.01) and 47.2% ± 12.8% (200 µM; *p* < 0.001; Table 1). Consequently, the relative amount of the SFAs PA and stearic acid (SA) decreased, with significant effects for both (200 µM; *p* < 0.01 and *p* < 0.05). Interestingly, *cis*-11-eicosenoic acid (gondoic acid; C20:1 *n*-9), the chain elongation product of OA, increased with higher doses of OA to 1.5% ± 0.4% (100 µM; *p* < 0.05) and 1.6% ± 0.6% (200 µM; *p* < 0.01). The relative amount of AA decreased after incubation with at least 100 µM OA to 0.3% ± 0.1% (100 µM, 200 µM; *p* < 0.01).

As AA is released from cellular PL pools upon cell stimulation (e.g., LPS), we analyzed whether the incubation of RAW264.7 macrophages with OA affects the FA composition of PLs. In our setup, we observed a decrease in PA in fractioned PLs after LPS stimulation and OA treatment (*p* < 0.05) as well as for POA (*p* < 0.05; Table 2). As for total lipids, the proportion of OA increased after incubation with OA or a combination of LPS and OA (*p* < 0.05). No significant alterations in the relative amount of gondoic acid and AA were observed. The relative amount of AdA in PL was below the detection limit.

### 3.6. Release of Arachidonic Acid in LPS-Stimulated Murine Macrophages Is Increased by OA

**Figure 5:**
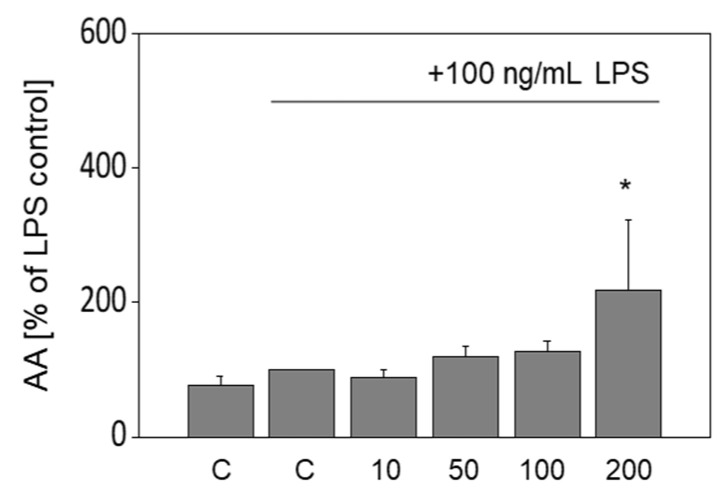
Effects of oleic acid (OA) on the release of arachidonic acid (AA). The release of AA in LPS-stimulated RAW264.7 macrophages is increased by incubation with OA. Cells were pre-incubated with serum-free medium, and the concentrations of OA as indicated for 4 h followed by a co-incubation of OA with 100 ng/mL LPS for an additional 20 h. Untreated control samples (C) were cultured with serum-free medium for 4 h plus another 20 h; values of positive controls (LPS) were defined as 100%. Data are shown as mean levels ± standard deviation of three independent biological replicates. Significant differences compared to the LPS control (* *p* < 0.05) were obtained by ordinary one-way ANOVA test.

It appeared possible that the increased PGE_2_ formation in OA-treated RAW264.7 cells could be due to elevated levels of AA as substrate. Thus, free AA was analyzed in cell culture supernatants of OA-treated RAW264.7 macrophages. While LPS did not induce the liberation of AA, 200 µM OA significantly augmented the release of AA in the supernatant to 218.9% ± 104.8% (*p* < 0.05). Lower concentrations of OA showed no significant effect (Figure 5).

## 4. Discussion

Olive oil is widely known for its health-promoting effects, especially in inflammatory diseases [30]. The PREDIMED (Prevención con Dieta Mediterránea) trial investigated Mediterranean dietary patterns with one patient group that consumed a defined amount of EVOO in addition to a Mediterranean diet. Although the PREDIMED study has its weaknesses [2], the EVOO-rich Mediterranean diet resulted in a significant reduction in cardiovascular events [31]. However, the molecular mechanisms behind the beneficial effects of EVOO are not yet fully understood.

We aimed to unravel the molecular mechanisms behind the anti-inflammatory properties of the lipophilic components of EVOO. Hence, we investigated the effects of these components on murine RAW 264.7 macrophages that are independent of the widely studied polyphenols [32,33,34]. We found that both OOE and unsaturated fatty acids (UFA) mediate anti-inflammatory effects on the LPS-induced inflammatory response. In our study, the EVOO has been hydrolyzed and purified by hexane extraction so that the obtained OOE consists almost completely of the FAs released from the triglycerides of the respective oils [18]. Depending on the food intake and fasting state, FA in human plasma can reach physiological concentrations up to 250 µM [35,36,37]. Hence, using 1 to 200 µM of FAs, we imitated postprandial physiological conditions in our experimental setup.

We focused on common inflammatory markers, namely iNos, Cox2, and selected cytokines (Il1β, Il6, Tnfα, and Il10). We found that OOE and OA reduced the expression of Cox2 and iNos, which have been shown for OA in previous studies [14,15]. In contrast, De Lima and co-workers reported an increased iNos protein expression after 12 h of incubation with OA in J774A.1 macrophages [38]. These different effects may be explained by the longer period of incubation (12 h vs. 24 h) and a different cell line (J774A.1 vs. RAW264.7) used here. Furthermore, OOE and OA mediated anti-inflammatory effects by partially significantly inhibiting the expression of Tnfα, Il1β, and Il6, as previously reported by others for OA [15,35].

To determine whether the major FAs of OOE, which differ in their degree of saturation and chain length, show different anti-inflammatory capacities, experiments were performed using the SFA PA, the MUFA OA, and the PUFA LA. Only OA and LA, as well as a mixture of PA, OA, and LA (80:10:10), showed significant inhibitory effects on Cox2 expression. These findings are in line with previously reported results using various cell types [15,39,40]. Furthermore, the LPS-induced cytokine expression was differentially affected by these FAs depending on their saturation and chain length. The saturated PA was less effective in reducing the LPS-induced upregulation of Tnfα, Il1β, Il6, and Il10 than the UFAs. The observed decrease in the proinflammatory Tnfα explains the reduced mRNA expression of the anti-inflammatory Il10 since the expression of Il10 is strongly induced by Tnfα [41]. De Lima et al. measured the time-dependent impact of PA on Tnfα expression in J774A.1 cells and found a decrease in expression followed by an increase over time [42]. In line with our results, other groups reported anti-inflammatory effects of UFA on cytokine expression or secretion [43,44,45]. Interestingly, Jin et al. described a boosting effect of PA on LPS inflammatory signaling in RAW264.7 cells [46]. Further, they studied antagonizing effects of UFA using OA and LA on LPS-PA-induced inflammation. While the combination of LPS, PA, and OA or LA slightly decreased the secretion of Il6, docosahexaenoic acid (DHA) significantly blocked LPS-PA-induced inflammation. Omega-3 (n3) PUFAs such as DHA are well-known for their anti-inflammatory properties [46]. Therefore, it can be assumed that antagonistic effects are triggered more effectively by n3-PUFA compared to OA. In our study, OA alone and OOE showed comparable effects on LPS-induced inflammation, which is in line with results reported by Jin et al. The interaction of FAs with classical inflammatory processes and with other FAs is an exciting starting point for further experimental investigations.

LPS-induced formation of nitric oxide was reduced by OOE, PA, OA, LA, and the FA mixture. In accordance with previous findings [14,15], we found a concentration-dependent effect of OA on the LPS-induced formation of nitric oxide. While only 10 µM of OA led to a significant increase in nitric oxide, higher concentrations significantly decreased nitric oxide formation. Similarly, De Lima et al., in turn, observed a notable increase in nitric oxide formation in J774A.1 macrophages for concentrations up to 100 µM OA and a decrease with 200 µM OA. The authors assumed cytotoxic effects for the higher doses that should be responsible for the lowering effect [38]. To exclude differences in the experimental setup, we conducted analyses using different concentrations (5 or 200 µM) of OA in the presence and absence of 100 ng/mL LPS in J774A.1 macrophages. We found a similar inhibition of nitric oxide formation (data not shown) as reported by De Lima et al. [38]. However, we tested cell viability in RAW264.7 up to 200 mM OA but observed no effect of OA on cell viability for the concentrations used in our experiments. The novelty of our study is the investigation of the lipophilic extract of olive oil. For the first time, we showed the anti-inflammatory effects of OOE on the LPS-induced formation of nitric oxide. In previous studies, we showed comparable effects of lipophilic nut extracts with OOE-like FA composition [18]. Indeed, the same concentration (200 µM) and processing (lipophilic extraction) resulted in reduced formation of nitric oxide in murine RAW264.7 macrophages. Thus, the composition of the extracts, whether from olive oil or from nuts, modulate LPS-induced inflammatory response. However, nut extracts, as well as commercial olive oil extracts (Figure 3B), showed slight variations in nitric oxide formation in comparison to OA, most likely due to antagonistic effects.

**Figure 6:**
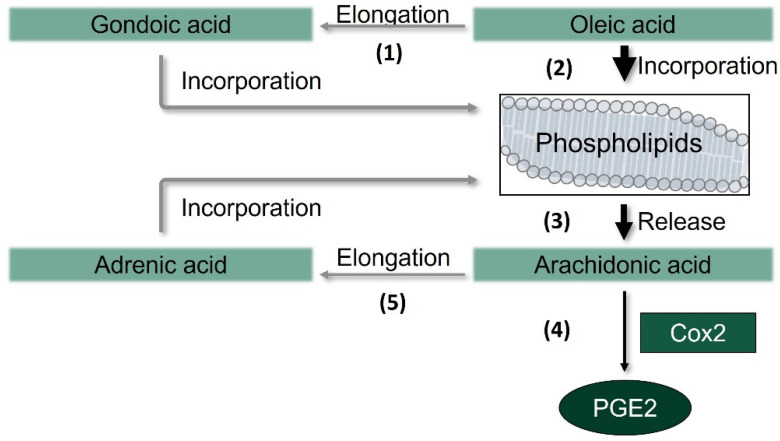
Potential mechanism of the elongation and incorporation of fatty acids in phospholipids and the formation of prostaglandin (PG) E_2_ by cyclooxygenase (Cox) 2 after incubation with oleic acid in RAW264.7 macrophages. Oleic acid is elongated to gondoic acid (**1**) or directly incorporated into the phospholipids (**2**), leading to a net release of arachidonic acid (AA) (**3**), a substrate of Cox2. As substrate availability increases, AA can either be converted by Cox2 to prostanoids such as PGE_2_ (**4**) or elongated to adrenic acid (**5**).

Stimulation with LPS significantly increases PGE_2_, the proinflammatory signaling molecule formed by Cox isoenzymes [47]. Interestingly, the LPS-induced release of PGE_2_ was augmented by co-incubation with OOE, LA (by trend), and the FA mixture, contrary to the drop of LPS-induced Cox2 mRNA and protein expression. To unravel the molecular mechanisms underlying this discrepancy, we investigated the effects of OA on PGE_2_ production in more detail and observed a concentration-dependent increase in the LPS-induced formation of PGE_2_. This effect is likely explained by changes in the compositions of cellular PL following uptake of OA (Figure 6).

Upon uptake into the cells, UFAs are incorporated into PLs of cellular membranes [48] by lysophospholipid acyltransferases isoenzymes, which have varying preferences for acyl-CoA substrates and overlapping specificities [49]. We postulate that OA and its elongation product gondoic acid compete with AA for being activated as CoA ester by acyl-CoA synthetases and being incorporated into PLs by lysophospholipid acyltransferases. The remodeling pathway, which continuously releases sn-2 fatty acids from PLs and re-incorporates them, seems, in consequence, to shift the balance from phospholipid-bound AA toward OA, thereby resulting in a net release of AA. This fatty acid is then either converted by Cox2 to respective lipid mediators such as PGE_2_ or elongated to adrenic acid. Tsai et al. reported similar results but investigated the PL composition of RAW264.7 macrophages after incubation with the *n*-3 PUFA juniperonic acid (C20:4), which, however, induced Cox2 protein expression [50].

## 5. Conclusions

In conclusion, OOE and OA inhibit the LPS-induced expression and formation of inflammatory mediators in murine macrophages. On the other hand, the formation of PGE_2_ and release of AA were significantly increased, and the FA composition of total cells as well as of the PL fraction changed after treatment with OA. We postulate that OA (i) substitutes for AA, (ii) is incorporated, either directly or after elongation to gondoic acid, into membrane PLs at the expense of AA, and (iii) thus enables the efficient conversion of AA to PGE_2_ despite impaired Cox2 expression. Interestingly, OA alone and OOE showed similar results for most parameters studied, although OOE contains next to the monounsaturated OA a variety of saturated and unsaturated FAs. These different classes of FA are known for different effects. Therefore, FAs contained in OOE could have agonistic and antagonistic effects that eventually balance each other. Through these mechanisms, the lipophilic fraction of EVOO contributes to both anti- and proinflammatory events in macrophages. Next to the lipophilic fraction, other components of olive oil, such as antioxidants, most likely strongly contribute to the well-known rather anti-inflammatory effect of EVOO. Further studies are needed to determine the physiological relevance of these findings in vivo.

## Figures and Tables

**Table 1:** Fatty acid composition (% of total FAME ^1^ as means ± S.D.) of RAW264.7-cells incubated with 0–200 µM OA compared to LPS-treated controls.

FAME	OA (µM)					
	0	0	10	50	100	200
	LPS (0.1 µg/mL)					
C16:0 (PA) ^2^	28.1 ± 4.8	32.0 ± 1.0	28.9 ± 1.9	25.0 ± 3.0	21.4 ± 7.6	16.3 ± 5.7 **
C16:1 (POA) ^3^	2.2 ± 0.5	2.2 ± 0.2	1.9 ± 0.3	0.8 ± 0.0 ***	0.6 ± 0.1 ***	0.5 ± 0.1 ***
C18:0 (SA) ^4^	28.0 ± 9.8	32.5 ± 2.7	28.1 ± 1.7	26.1 ± 2.3	22.9 ± 2.8	20.9 ± 4.8 *
C18:1 (OA) ^5^	18.5 ± 3.1	15.6 ± 1.7	20.9 ± 2.4	30.3 ± 2.8	40.2 ± 9.8 **	47.2 ± 12.8 ***
C18:2 (LA) ^6^	1.6 ± 0.5	1.3 ± 0.4	1.4 ± 0.2	1.3 ± 0.3	1.4 ± 0.7	1.9 ± 1.8
C20:1 (GA) ^7^	0.7 ± 0.1	0.5 ± 0.1	0.8 ± 0.2	1.2 ± 0.2	1.5 ± 0.4 *	1.6 ± 0.6 **
C20:4 (AA) ^8^	1.4 ± 0.4	1.0 ± 0.1	1.0 ± 0.1	0.6 ± 0.1	0.3 ± 0.1 **	0.3 ± 0.1 **
C22:4 (AdA) ^9^	0.0 ± 0.0	0.2 ± 0.0	0.2 ± 0.1	0.2 ± 0.0	0.2 ± 0.1	0.2 ± 0.1

^1^ FAME, fatty acid methyl esters. ^2^ PA, palmitic acid. ^3^ POA, palmiolic acid, ^4^ SA, stearic acid. ^5^ OA, oleic acid. ^6^ LA, linoleic acid. ^7^ GA, gondoic acid, ^8^ AA, arachidonic acid, ^9^ AdA, adrenic acid. * *p* < 0.05; ** *p* < 0.01; *** *p* < 0.001; ordinary one-way ANOVA test, *n* = 3.

**Table 2:** Fatty acid composition (% of total FAME ^1^ as means ± S.D) of phospholipids of murine RAW264.7 macrophages after incubation with 200 µM OA compared to LPS-treated control cells.

FAME	0 µM OA	200 µM OA	0 µM OA + LPS (0.1 µg/mL)	200 µM OA + LPS (0.1 µg/mL)
C16:0 (PA) ^2^	19.4 ± 1.7	14.0 ± 3.2 *	20.7 ± 6.5	12.8 ± 3.8 *
C16:1 (POA) ^3^	1.6 ± 0.7	0.9 ± 0.6	1.5 ± 0.4	0.7 ± 0.3 *
C18:0 (SA) ^4^	13.4 ± 2.6	9.4 ± 1.5	13.7 ± 4.0	9.9 ± 4.7
C18:1 (OA) ^5^	13.9 ± 5.2	22.4 ± 5.3 *	12.6 ± 5.1	33.3 ± 12.3 *
C18:2 (LA) ^6^	2.0 ± 1.5	2.4 ± 0.6 *	1.3 ± 0.5	1.6 ± 0.6
C20:1 (GA) ^7^	0.8 ± 0.3	1.7 ± 1.0	2.1 ± 4.0	1.2 ± 0.6
C20:4 (AA) ^8^	0.7 ± 0.5	0.6 ± 0.6	0.3 ± 0.4	0.2 ± 0.2

^1^ FAME, fatty acid methyl esters. ^2^ PA, palmitic acid. ^3^ POA, palmiolic acid, ^4^ SA, stearic acid. ^5^ OA, oleic acid. ^6^ LA, linoleic acid. ^7^ gondoic acid, ^8^ AA, arachidonic acid. * *p* < 0.05; ordinary one-way ANOVA test, *n* = 6.

## Data Availability

The datasets used and/or analyzed during the current study are available from the corresponding author upon reasonable request.

## Supplementary Materials

The following are available online at https://www.mdpi.com/article/10.3390/nu13124437/s1, Table S1: Concentration of major fatty acids in OOE; Table S2: PCR primers used in this study. In each case, forward and reverse primers are located in different exons; Table S3: MTT cell viability of RAW264.7 cells (%); Table S4: Fatty acid composition (% of total FAME) of olive oil extracts.

## References

[1] Estruch R., Ros E., Salas-Salvadó J., Covas M.-I., Corella D., Arós F., Gómez-Gracia E., Ruiz-Gutiérrez V., Fiol M., Lapetra J. (2013). Primary Prevention of Cardiovascular Disease with a Mediterranean Diet. N. Engl. J. Med..

[2] Agarwal A., Ioannidis J.P.A. (2019). PREDIMED trial of Mediterranean diet: Retracted, republished, still trusted?. BMJ.

[3] Finicelli M., Squillaro T., Di Cristo F., Di Salle A., Melone M.A.B., Galderisi U., Peluso G. (2019). Metabolic syndrome, Mediterranean diet, and polyphenols: Evidence and perspectives. J. Cell. Physiol..

[4] Schwingshackl L., Christoph M., Hoffmann G.F. (2015). Effects of Olive Oil on Markers of Inflammation and Endothelial Function—A Systematic Review and Meta-Analysis. Nutrients.

[5] Shen J., Wilmot K.A., Ghasemzadeh N., Molloy D.L., Burkman G., Mekonnen G., Gongora M.C., Quyyumi A.A., Sperling L.S. (2015). Mediterranean Dietary Patterns and Cardiovascular Health. Annu. Rev. Nutr..

[6] Buckley C.D., Gilroy D., Serhan C.N. (2014). Proresolving Lipid Mediators and Mechanisms in the Resolution of Acute Inflammation. Immunity.

[7] Calder P.C. (2011). Fatty acids and inflammation: The cutting edge between food and pharma. Eur. J. Pharmacol..

[8] Hamidzadeh K., Christensen S.M., Dalby E., Chandrasekaran P., Mosser D.M. (2017). Macrophages and the Recovery from Acute and Chronic Inflammation. Annu. Rev. Physiol..

[9] Ait-Oufella H., Taleb S., Mallat Z., Tedgui A. (2011). Recent Advances on the Role of Cytokines in Atherosclerosis. Arter. Thromb. Vasc. Biol..

[10] Aktan F. (2004). iNOS-mediated nitric oxide production and its regulation. Life Sci..

[11] Simmons D.L., Botting R.M., Hla T. (2004). Cyclooxygenase Isozymes: The Biology of Prostaglandin Synthesis and Inhibition. Pharmacol. Rev..

[12] Rastrelli L., Passi S., Ippolito F., Vacca G., De Simone F. (2002). Rate of Degradation of α-Tocopherol, Squalene, Phenolics, and Polyunsaturated Fatty Acids in Olive Oil during Different Storage Conditions. J. Agric. Food Chem..

[13] Carluccio M.A., Massaro M., Bonfrate C., Siculella L., Maffia M., Nicolardi G., Distante A., Storelli C., Caterina R.D. (1999). Oleic Acid Inhibits Endothelial Activation: A Direct Vascular Antiatherogenic Mechanism of a Nutritional Component in the Mediterranean Diet. Arterioscler. Thromb. Vasc. Biol..

[14] Oh Y.T., Lee J.Y., Lee J., Kim H., Yoon K.-S., Choe W., Kang I. (2009). Oleic acid reduces lipopolysaccharide-induced expression of iNOS and COX-2 in BV2 murine microglial cells: Possible involvement of reactive oxygen species, p38 MAPK, and IKK/NF-κB signaling pathways. Neurosci. Lett..

[15] Chang C.F., Chau Y.P., Ni Kung H., Lu K.S. (2012). The lipopolysaccharide-induced pro-inflammatory response in RAW264.7 cells is attenuated by an unsaturated fatty acid–bovine serum albumin complex and enhanced by a saturated fatty acid–bovine serum albumin complex. Inflamm. Res..

[16] De Santis S., Liso M., Verna G., Curci F., Milani G., Faienza M., Franchini C., Moschetta A., Chieppa M., Clodoveo M. (2021). Extra Virgin Olive Oil Extracts Modulate the Inflammatory Ability of Murine Dendritic Cells Based on Their Polyphenols Pattern: Correlation between Chemical Composition and Biological Function. Antioxidants.

[17] Teres S., Barceló-Coblijn G., Benet M., Alvarez R., Bressani R., Halver J.E., Escriba P.V. (2008). Oleic acid content is responsible for the reduction in blood pressure induced by olive oil. Proc. Natl. Acad. Sci. USA.

[18] Müller A.K., Schmölz L., Wallert M., Schubert M., Schlörmann W., Glei M., Lorkowski S. (2019). In Vitro Digested Nut Oils Attenuate the Lipopolysaccharide-Induced Inflammatory Response in Macrophages. Nutrients.

[19] Krist S. (2013). Lexikon der pflanzlichen Fette und Öle.

[20] Bligh E.G., Dyer W.J. (1959). A RAPID METHOD OF TOTAL LIPID EXTRACTION AND PURIFICATION. Can. J. Biochem. Physiol..

[21] Hošek J., Závalová V., Kollár P. (2010). Effect of solvent on cytotoxicity and bioavailability of fatty acids. Immunopharmacol. Immunotoxicol..

[22] Banning A., Florian S., Deubel S., Thalmann S., Müller-Schmehl K., Jacobasch G., Brigelius-Flohé R. (2008). GPx2 Counteracts PGE2Production by Dampening COX-2 and mPGES-1 Expression in Human Colon Cancer Cells. Antioxidants Redox Signal..

[23] Wallert M., Schmölz L., Koeberle A., Krauth V., Glei M., Galli F., Werz O., Birringer M., Lorkowski S. (2015). α-Tocopherol long-chain metabolite α-13′-COOH affects the inflammatory response of lipopolysaccharide-activated murine RAW264.7 macrophages. Mol. Nutr. Food Res..

[24] Stolle K., Schnoor M., Fuellen G., Spitzer M., Cullen P., Lorkowski S. (2007). Cloning, genomic organization, and tissue-specific expression of the RASL11B gene. Biochim. Biophys. Acta.

[25] Stolle K., Schnoor M., Fuellen G., Spitzer M., Engel T., Spener F., Cullen P., Lorkowski S. (2005). Cloning, cellular localization, genomic organization, and tissue-specific expression of the TGFβ1-inducible SMAP-5 gene. Gene.

[26] Schmölz L., Wallert M., Lorkowski S. (2017). Optimized incubation regime for nitric oxide measurements in murine macrophages using the Griess assay. J. Immunol. Methods.

[27] Schaible A., Koeberle A., Northoff H., Lawrenz B., Weinigel C., Barz D., Werz O., Pergola C. (2013). High capacity for leukotriene biosynthesis in peripheral blood during pregnancy. Prostaglandins Leukot. Essent. Fat. Acids.

[28] Werz O., Gerstmeier J., Libreros S., De La Rosa X., Werner M., Norris P., Chiang N., Serhan C.N. (2018). Human macrophages differentially produce specific resolvin or leukotriene signals that depend on bacterial pathogenicity. Nat. Commun..

[29] Pein H., Ville A., Pace S., Temml V., Garscha U., Raasch M., Alsabil K., Viault G., Dinh C.-P., Guilet D. (2018). Endogenous metabolites of vitamin E limit inflammation by targeting 5-lipoxygenase. Nat. Commun..

[30] Piroddi M., Albini A., Fabiani R., Giovannelli L., Luceri C., Natella F., Rosignoli P., Rossi T., Taticchi A., Servili M. (2016). Nutrigenomics of extra-virgin olive oil: A review. BioFactors.

[31] Estruch R., Ros E., Salas-Salvadó J., Covas M.-I., Corella D., Arós F., Gómez-Gracia E., Ruiz-Gutiérrez V., Fiol M., Lapetra J. (2018). Primary Prevention of Cardiovascular Disease with a Mediterranean Diet Supplemented with Extra-Virgin Olive Oil or Nuts. N. Engl. J. Med..

[32] Cárdeno A., Sánchez-Hidalgo M., Aparicio-Soto M., Sánchez-Fidalgo S., Alarcón-De-La-Lastra C. (2014). Extra virgin olive oil polyphenolic extracts downregulate inflammatory responses in LPS-activated murine peritoneal macrophages suppressing NFkB and MAPK signalling pathways. Food Function.

[33] Lu J., Huang G., Wang Z., Zhuang S., Xu L., Song B., Xiong Y., Guan S. (2013). Tyrosol exhibits negative regulatory effects on LPS response and endotoxemia. Food Chem. Toxicol..

[34] Casas R., Estruch R., Sacanella E. (2018). The Protective Effects of Extra Virgin Olive Oil on Immune-mediated Inflammatory Responses. Endocr. Metab. Immune Disord. Drug Targets.

[35] Lu G., Morinelli T.A., Meier K.E., Rosenzweig S.A., Egan B.M. (1996). Oleic Acid–Induced Mitogenic Signaling in Vascular Smooth Muscle Cells. Circ. Res..

[36] Jump D.B., Clarke S.D. (1999). Regulation of gene expression by dietary fat. Annu. Rev. Nutr..

[37] Clore J.N., Allred J., White D., Li J., Stillman J. (2002). The role of plasma fatty acid composition in endogenous glucose production in patients with type 2 diabetes mellitus. Metabolism.

[38] De Lima T.M., Lima L.D.S., Scavone C., Curi R. (2006). Fatty acid control of nitric oxide production by macrophages. FEBS Lett..

[39] Komatsu W. (2003). Docosahexaenoic acid suppresses nitric oxide production and inducible nitric oxide synthase expression in interferon-? plus lipopolysaccharide-stimulated murine macrophages by inhibiting the oxidative stress. Free. Radic. Biol. Med..

[40] Lee J.Y., Sohn K.H., Rhee S.H., Hwang D. (2001). Saturated fatty acids, but not unsaturated fatty acids, induce the expression of cyclooxygenase-2 mediated through Toll-like receptor 4. J. Biol. Chem..

[41] Wanidworanun C., Strober W. (1993). Predominant role of tumor necrosis factor-alpha in human monocyte IL-10 synthesis. J. Immunol..

[42] de Lima-Salgado T.M., Alba-Loureiro T.C., do Nascimento C.S., Nunes M.T., Curi R. (2011). Molecular mechanisms by which saturated fatty acids modulate TNF-alpha expression in mouse macrophage lineage. Cell Biochem. Biophys..

[43] Honda K.L., Lamon-Fava S., Matthan N.R., Wu D., Lichtenstein A.H. (2015). EPA and DHA Exposure Alters the Inflammatory Response but not the Surface Expression of Toll-like Receptor 4 in Macrophages. Lipids.

[44] Weldon S.M., Mullen A.C., Loscher C.E., Hurley L.A., Roche H.M. (2007). Docosahexaenoic acid induces an anti-inflammatory profile in lipopolysaccharide-stimulated human THP-1 macrophages more effectively than eicosapentaenoic acid. J. Nutr. Biochem..

[45] Zhao G., Etherton T.D., Martin K.R., Heuvel J.P.V., Gillies P.J., West S.G., Kris-Etherton P.M. (2005). Anti-inflammatory effects of polyunsaturated fatty acids in THP-1 cells. Biochem. Biophys. Res. Commun..

[46] Jin J., Lu Z., Li Y., Cowart L.A., Lopes-Virella M.F., Huang Y. (2018). Docosahexaenoic acid antagonizes the boosting effect of palmitic acid on LPS inflammatory signaling by inhibiting gene transcription and ceramide synthesis. PLoS ONE.

[47] Tsatsanis C., Androulidaki A., Venihaki M., Margioris A.N. (2006). Signalling networks regulating cyclooxygenase-2. Int. J. Biochem. Cell Biol..

[48] Calder P.C. (2008). The relationship between the fatty acid composition of immune cells and their function. Prostaglandins, Leukot. Essent. Fat. Acids.

[49] Kita Y., Shindou H., Shimizu T. (2019). Cytosolic phospholipase A(2) and lysophospholipid acyltransferases. Biochim. Biophys Acta Mol Cell Biol Lipids.

[50] Tsai P.J., Huang W.C., Lin S.W., Chen S.N., Shen H.J., Chang H., Chuang L.T. (2018). Juniperonic Acid Incorporation into the Phospholipids of Murine Macrophage Cells Modulates Pro-Inflammatory Mediator Production. Inflammation.

