# Supplementary material for: Olive Oil Extracts and Oleic Acid Attenuate the LPS-Induced Inflammatory Response in Murine RAW264.7 Macrophages but Induce the Release of Prostaglandin E2
Source: Nutrients. 2021 Dec 11;13(12):4437. doi: 10.3390/nu13124437 (PMC8703532; doi:10.3390/nu13124437)
Supplement: Supplementary file 1 [file nutrients-13-04437-s001.zip › nutrients-1485079-supplementary.pdf]

Olive oil extracts and oleic acid attenuate the LPS-induced inflammatory response in murine RAW264.7 macrophages but induce the release of prostaglandin E_2

Anke Katharina Müller[1,2], Franziska Albrecht[1], Carsten Rohrer[1,2], Andreas Koeberle[4,5], Oliver Werz[5], Wiebke Schlörmann[2,3], Michael Glei[2,3], Stefan Lorkowski[1,2], Maria Wallert[1,2*]

Supplementary Table S1: Concentration of major fatty acids in OOE[1].

Fatty acid	Concentration [mol/l]
C16:0	0.379
C16:1c9	0.022
C18:0	0.076
C18:1c9	2.517
C18:2c9,12	0.180
C18:3c9,12,15	0.017
C20:0	0.010
**Total**	**3.200**

[1] OOE, olive oil extract.

Supplementary Table S2: PCR primers used in this study. In each case, forward and reverse primers are located in different exons.

mRNA	mRNA name	GenBank accession no.	Forward primer	Reverse primer	Amplicon size [bp]
Cox2	Cyclooxygenase 2	NM_011198	TCCCTGAAGCCGTACACATCA	TGGACGAGGTTTTTCCACCA	132
Il1β	Interleukin 1 beta	NM_008361.3	TGAAGTTGACGGACCCCAAA	CAGCCACAATGAGTGATACTGCC	140
Il6	Interleukin 6	NM_031168.1	TCAATTCCAGAAACCGCTATGAA	GGAAGGCCGTGGTTGTCAC	94
Il10	Interleukin 10	NM_010548.2	AAATAAGAGCAAGGCAGTGGAGC	TCATTCATGGCCTTGTAGACACC	81
iNos	Inducible nitric oxide synthases	NM_010927.3	GAGCGAGGAGCAGGTGGAA	CCATAGGAAAAGACTGCACCGA	90
Ppib	Peptidylprolyl isomerase B	NM_011149.2	AAACAGCAAGTTCCATCGTGTCAT	GAAGCGCTCACCATAGATGCTCT	103
Tnfα	Tumor necrosis factor α	NM_013693	AGAAACACAAGATGCTGGGACAGT	CCTTTGCAGAACTCAGGAATGG	46

Supplementary Table S3: MTT cell viability of RAW264.7 cells (%)

Concentration (μM)	OOE[1]	OA[2]
0	100 ± 0	100 ± 0
1	106.9 ± 2.9	95.1 ± 6.9
5	116.2 ± 7.8	101.6 ± 8.5
10	116.0 ± 6.2	100.5 ± 10.6
50	112.8 ± 4.9	105.3 ± 10.2
100	114.1 ± 5.9	109.4 ± 6.9
200	127.7 ± 9.6	115.1 ± 9.0

[1] OOE, olive oil extract; [2] OA, Oleic acid

Supplementary Table S4: Fatty acid composition (% of total FAME[1]) of olive oil extracts

	O 1	O2	O3	O4	O5
C16:0	9.4	10.3	10.6	14.6	12.2
C18:0	2.9	2.4	2.3	2.1	2.3
C18:1c9	81.3	78.0	77.8	66.9	72.8
C18:2 n-6 (LA)[2]	3.3	5.6	5.5	11.4	8.4
C-18:3 n-3 (ALA)[3]	0.5	0.5	0.5	0.5	0.5

[1] FAME, fatty acid methyl esters. [2] LA, linoleic acid. [3] ALA, α-linolenic acid.
